# Interobserver study on histologic features of idiopathic non-cirrhotic portal hypertension

**DOI:** 10.1186/s13000-020-01049-0

**Published:** 2020-10-23

**Authors:** Michel Kmeid, Chunlai Zuo, Stephen M. Lagana, Won-Tak Choi, Jingmei Lin, Zhaohai Yang, Xiuli Liu, Maria Westerhoff, M. Isabel Fiel, Kajsa Affolter, Eun-Young K. Choi, Hwajeong Lee

**Affiliations:** 1 Department of Pathology, Albany Medical Center, 47 New Scotland Ave., MC81, Albany, NY 10032 USA; 2 Department of Pathology, Columbia University, New York, NY 10027 USA; 3 Department of Pathology, University of California San Francisco, San Francisco, CA 46202 USA; 4 Department of Pathology, Indiana University, Indianapolis, IN 47405 USA; 5 Department of Pathology, University of Pennsylvania, Philadelphia, PA 19104 USA; 6 Department of Pathology, University of Florida at Gainesville, Gainesville, FL 32608 USA; 7 Department of Pathology, University of Michigan, Ann Arbor, MI 48109 USA; 8 Department of Pathology, Mount Sinai Medical Center, New York, NY 10029 USA; 9 Department of Pathology, University of Utah, Salt Lake City, UT 84132 USA

**Keywords:** Portal hypertension, Porto-sinusoidal vascular disease, Interobserver

## Abstract

**Background:**

Histologic features of idiopathic non-cirrhotic portal hypertension (INCPH) may overlap with those without INCPH. Recently, these features have been recognized as part of the larger spectrum of porto-sinusoidal vascular disease (PSVD). We assessed interobserver agreement on histologic features that are commonly associated with INCPH and studied whether a provision of relevant clinical history improves interobserver agreement.

**Methods:**

The examined histologic features include lobular (such as anisocytosis, nodular regeneration, sinusoidal dilatation, increased parenchymal draining veins, and incomplete fibrous septa) and portal tract changes (such as paraportal shunting vessel(s), portal tract remnant, increased number of portal vessels, and obliterative portal venopathy). Thirty-four archived liver samples from patients with (group A) and without (group B) INCPH were retrieved. A total of 90 representative images of lobules (L) and portal tracts (P) were distributed among 9 liver pathologists blinded to true clinical history. Each pathologist answered multiple choice questions based on the absence (Q1) or presence (Q2) of clinical history of portal hypertension. Fleiss’ kappa coefficient analysis (unweighted) was performed to assess interobserver agreement on normal versus abnormal diagnosis, in L and P, based on Q1 and Q2.

**Results:**

The kappa values regarding normal versus abnormal diagnosis were 0.24, 0.24, 0.18 and 0.18 for L-Q1, L-Q2, P-Q1, and P-Q2, respectively. With true clinical history provided, the kappa values were L- 0.32, P-0.17 for group A and L-0.12, P-0.14 for group B. Four pathologists changed their assessments based on the provided history. Interobserver agreement on the interpretation of L and P as normal versus abnormal was slight to fair regardless of provision of clinical history.

**Conclusions:**

Our findings indicate that the histologic features of INCPH/PSVD are not limited to patients with portal hypertension and are subject to significant interobserver variation.

**Supplementary information:**

The online version contains supplementary material available at 10.1186/s13000-020-01049-0.

## Background

Idiopathic non-cirrhotic portal hypertension (INCPH) is a clinical disorder manifested by signs of portal hypertension in the absence of cirrhosis [1–3]. The diagnosis is made after excluding known non-cirrhotic causes of portal hypertension, such as infiltrative liver diseases (i.e., sarcoidosis), schistosomiasis, portal vein or splenic vein thrombosis, and Budd-Chiari syndrome, as well as any forms of chronic liver disease [2]. Previously, different terminologies were used for this entity encompassing non-cirrhotic portal fibrosis, idiopathic portal hypertension, hepatoportal sclerosis, obliterative portal venopathy, and partial nodular transformation. The term INCPH was first proposed by Shouten et al. in 2011 [1, 3]. Although the exact etiology of INCPH remains unknown, it appears to develop as a result of the occlusion of small intrahepatic branches of the portal vein leading to increased portal flow resistance [2, 4]. Commonly observed histologic features include lobular (such as nodular regeneration, sinusoidal dilatation, increased parenchymal draining veins, and incomplete fibrous septa) and portal tract changes (such as paraportal shunting vessel(s), portal tract remnant, increased number of portal vessels, and obliterative portal venopathy) [1–7]. Indeed, obliterative portal venopathy is often regarded as the hallmark feature of INCPH [3].

However, it has been increasingly recognized that INCPH-like histologic features can be seen in liver samples without evidence of portal hypertension, including in liver biopsies obtained during cholecystectomy or gastric bypass, fatty liver disease [8], regressed fibrosis [9], and normal “control” liver [10]. As such, the clinical significance of individual or any combination of histologic features that can be seen in INCPH remains speculative in daily liver pathology practice. Also, it is unknown whether such histologic findings can be consistently recognized by pathologists.

To broaden the definitional spectrum of INCPH and capture the pre-portal hypertension phase of INCPH, the Vascular Liver Disease Interest Group (VALDIG) recently introduced the term “porto-sinusoidal vascular disease” (PSVD) [11]. This newly proposed term attempts to overcome the shortcomings of previous terminologies by including patients at earlier stages without evidence of portal hypertension. Under this new classification, clinical history of portal hypertension is no longer required for the diagnosis of PSVD, as long as “specific” histological features (including obliterative portal venopathy, nodular regenerative hyperplasia (NRH), and incomplete septal cirrhosis (ISC)) are present.

However, this broader diagnostic approach to this relatively poorly understood entity raises two specific concerns. First, it places emphasis primarily on histomorphologic features for the diagnosis (i.e., obliterative portal venopathy, NRH, and ISC), despite the fact that these features can be subtle and lack adequately validated defining criteria. Second, the nonrestrictive nature of this new classification broadens the aspects of this disorder to the point where biopsies that fulfill the criteria for PSVD may not identify patients with a clinical disease. In fact, to date, the clinical significance of individual histologic features associated with INCPH/PSVD in the absence of appropriate clinical context is largely unknown. As noted above, these features may be discovered incidentally without portal hypertension, and the risk of progression and long-term outcome in this setting remain unexplored.

As such, in this study, we evaluated whether liver pathologists can reach consensus on individual histologic features that are typically seen in INCPH. We also studied whether provision of clinical history of portal hypertension impacts their assessments of these histologic features. Furthermore, we applied the recently proposed histologic criteria for PSVD (i.e., obliterative portal venopathy, NRH, and ISC) to our study cases irrespective of clinical history and compared the frequencies of true INCPH and non-INCPH cases that would fulfill the PSVD criteria, based purely on histologic assessment.

## Methods

After approval from the Institutional Review Board at Albany Medical Center, 34 archived liver samples from patients with and without INCPH were retrieved. Liver samples with tumor, cirrhosis, advanced fibrosis, or significant steatosis were excluded. Group A (INCPH group) consisted of 15 hematoxylin and eosin (H&E)-stained liver samples (14 core needle biopsies and 1 wedge biopsy) from 12 patients (mean age: 46, range: 13–76 years, 8 males and 4 females) with portal hypertension. Group B (non-INCPH) consisted of 19 H&E-stained liver samples (16 core needle biopsies and 3 wedge biopsies) from 19 patients (mean age: 46, range: 16–74 years, 9 males and 10 females) without evidence of INCPH or portal hypertension. One needle biopsy from group A and three wedge biopsies from group B were subcapsular sampling, histologically. Ten of 19 group B cases had been used for previous studies [8, 9, 12]. The principal investigator (HL) selected additional 9 cases after assessing histologic features. The indications for liver biopsy in group B were abnormal liver function tests (*n* = 13), intraoperative fatty-appearing liver (*n* = 2), hyperammonemia (*n* = 1), evaluation for lung transplant (*n* = 1) and unknown (n = 2). Three group B patients had underlying liver diseases without advanced fibrosis: hereditary hemochromatosis (n = 1), HCV (n = 1), and alpha-1 antitrypsin deficiency status post liver transplant (n = 1). No liver disease was documented in the remaining group B patients. Follow-up (mean 38 months, range 1 to 84 months) was available in 14 of 19 group B patients. No patient developed portal hypertension at the end of the follow-up.

**Fig. 1:**
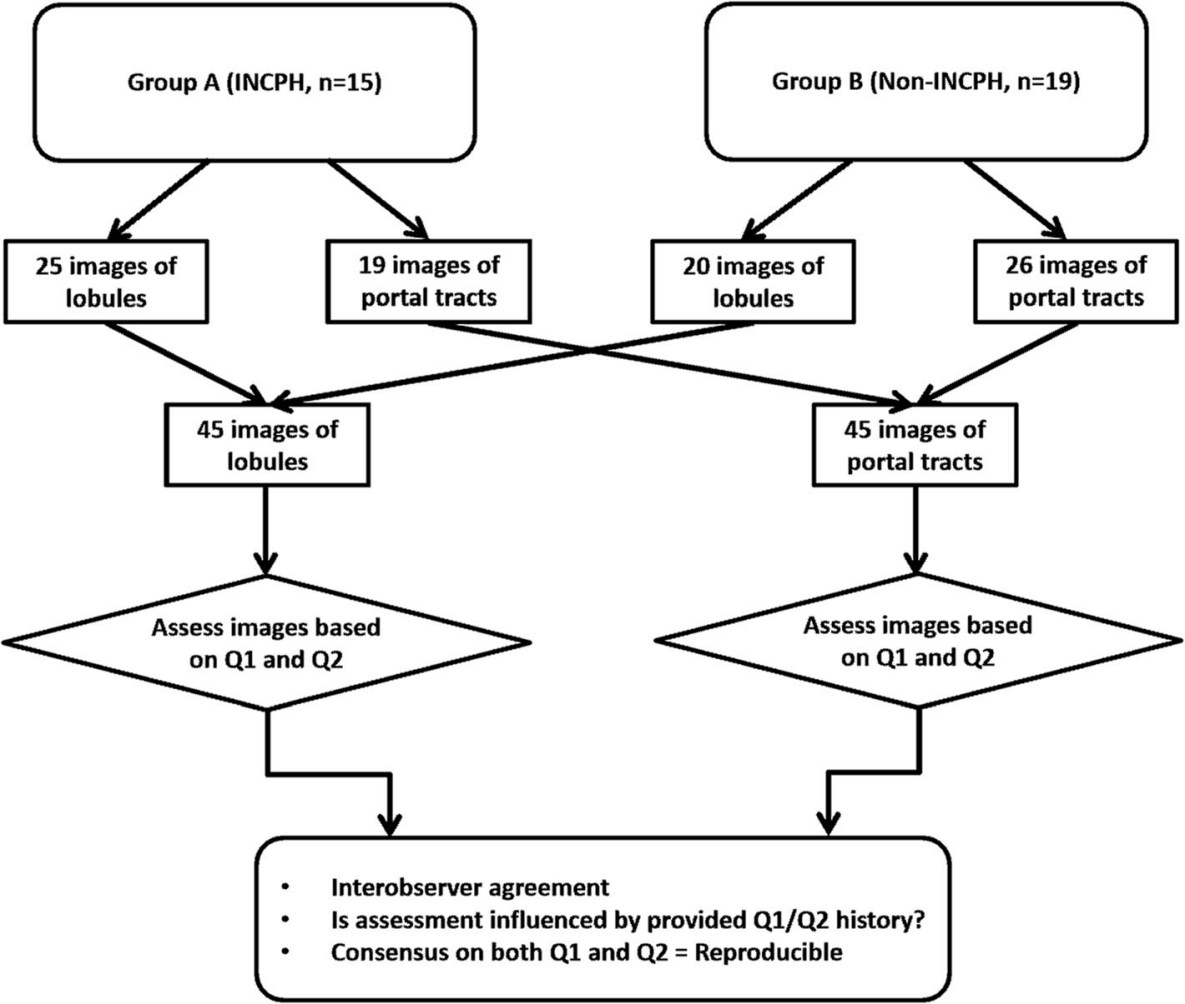
Study design. INCPH, idiopathic non-cirrhotic portal hypertension; Q1, no clinical history of portal hypertension; Q2, clinical history of portal hypertension

A total of 90 images of lobules and portal tracts were captured by the principal investigator to allow for both overall and focused assessment of lobular and portal tract changes. The images included 45 from the lobules (labeled as L1 to L45) and 45 from the portal tracts (labeled as P1 to P45), and then were distributed to 9 liver pathologists from 8 different institutions. The participants were blinded to the original diagnosis and clinical history (Fig. 1).

The pathologists were asked to evaluate each image under 2 scenarios: Q1: If there is no clinical history of portal hypertension, is this image of lobule or portal tract considered within normal limits or not? Q2: If there is a clinical history of portal hypertension (in other words, you start to consider a possibility of INCPH in this case), is this image of lobule or portal tract considered within normal limits or not? For the 90 images, the pathologists were asked to choose between “within normal limits” (choice a) and multiple choices (to choose at least one) if the image was considered abnormal, based on Q1 and Q2. The choices for abnormal lobular images were: (b) anisocytosis; (c) nodular regeneration; (d) sinusoidal dilatation; (e) increased parenchymal draining veins, and (f) incomplete fibrous septa. Anisocytosis was included as one of the answer choices to test its potential utility as a surrogate for NRH. The choices for abnormal portal images were: (b) paraportal shunting vessel(s); (c) portal tract remnant; (d) increased number of portal vessels, and (e) obliterative portal venopathy (Table 1).

**Table 1:** Questionnaires

Questions for L1 to L45 (Lobules)	Questions for P1 to P45 (Portal tracts)
Please review the lobules in the image.	Please review the portal tract in the image.
The images are taken at × 10.	The images are taken at ×20.
Please choose your answer based on your assessment of lobules only, not the portal tracts.	When more than one portal tracts are noted, review the one in the center of the image.
	Please choose your answer based on your assessment of portal tracts only, not the lobules.
Q1-L) If there is **NO** clinical history of portal hypertension and there is no advanced fibrosis (no bridging fibrosis or cirrhosis) in the sample, this lobule shows/is:	Q1-P) If there is **NO** clinical history of portal hypertension and there is no advanced fibrosis (no bridging fibrosis or cirrhosis) in the sample, this portal tract shows/is:
a. Within normal limits (single choice)	a. Within normal limits (single choice)
**OR**	**OR**
(b-f: multiple choice, you can choose one or more answers)	(b-e: multiple choice, you can choose one or more answers)
b. Anisocytosis	b. Paraportal shunting vessel(s)
c. Nodular regeneration	c. Portal tract remnant
d. Sinusoidal dilatation	d. Increased number of portal vessels
e. Increased parenchymal draining veins	e. Obliterative portal venopathy (phlebosclerosis)
f. Incomplete fibrous septa	
Q2-L) If there **IS** a clinical history of portal hypertension and there is no advanced fibrosis (no bridging fibrosis or cirrhosis) in the sample (in other words, you start to consider a possibility of INCPH in this case) this lobule shows/is:	Q2-P) If there **IS** a clinical history of portal hypertension and there is no advanced fibrosis (no bridging fibrosis or cirrhosis) in the sample (in other words, you start to consider a possibility of INCPH in this case), this portal tract shows/is:
a. Within normal limits (single choice)	a. Within normal limits (single choice)
**OR**	**OR**
(b-f: multiple choice, you can choose one or more answers)	(b-e: multiple choice, you can choose one or more answers)
b. Anisocytosis	b. Paraportal shunting vessel(s)
c. Nodular regeneration	c. Portal tract remnant
d. Sinusoidal dilatation	d. Increased number of portal vessels
e. Increased parenchymal draining veins	e. Obliterative portal venopathy (phlebosclerosis)
f. Incomplete fibrous septa	
Q3-L) Comment (optional)	Q3-P) Comment (optional)

*INCPH* idiopathic non-cirrhotic portal hypertension

Previously proposed diagnostic criteria for each of these lobular and portal changes were provided to participating pathologists along with the reference article containing representative microscopic images [13]. In addition, an article describing a detailed morphometric analysis of normal adult human liver was provided as a reference for normal liver [14]. The definitions are as follows: nodular regeneration - parenchymal micronodular transformation with central hyperplasia and peripheral atrophy without fibrosis; increased parenchymal draining veins - multiple dilated parenchymal veins clustered in the lobule; incomplete fibrous septa - thin, blindly ending septa; paraportal shunting vessel(s) - enlarged thin-walled vessels outside, but in close contact with the portal tract; portal tract remnant - a portal tract of which the size is smaller than twice the diameter of the bile duct; increased number of portal vessels - multiple small vascular channels in portal tract; obliterative portal venopathy - portal vein with a reduced lumen in a fibrotic portal tract [7, 10, 13].

When 6 or more pathologists chose the same answer for a certain image, the answer was considered as “consensus.” We counted the number of images that reached consensus in each category (L-Q1, L-Q2, P-Q1, and P-Q2). For each answer choice (L: a to e, P: a to f), we counted how many times a consensus was achieved for the specific answer choice, for Q1 and Q2, respectively. We then calculated the difference in the consensus number between Q1 and Q2 for each answer. For instance, if answer “b” is the consensus answer for 5 lobular images when Q1 was provided, and for 10 lobular images for Q2, the difference would be 10–5 = + 5. Thus, by provision of Q2 history (i.e., clinical history of portal hypotension), 5 additional images are agreed upon to represent “b” by ≥6 pathologists. The interpretation of the image was considered “reproducible” when consensus was reached on both Q1 and Q2, non-reproducible when the provision of Q1/Q2 history changed the consensus status (i.e., consensus to no consensus or vice versa), and divergent when no consensus was reached on both Q1 and Q2. Fleiss’ kappa statistic was used to assess the interobserver agreement on each image. We also evaluated if this agreement is impacted by clinical history (Fig. 1). Kappa (unweighted) values between 0.01–0.20 were considered slight, 0.21–0.40 fair, 0.41–0.60 moderate, 0.61–0.80 substantial, and 0.81–1.00 almost perfect agreement. Z-scores were then calculated and converted to a *p*-value. A p-value < 0.05 was considered statistically significant. Statistical analysis was performed using SPSS statistics online software.

Cases from INCPH (group A) and non-INCPH (group B) were re-assessed according to the recently proposed histologic criteria for PSVD by two authors (MK and HL) using whole slides. PSVD diagnosis can be rendered based on the presence of one or more histologic lesions, including obliterative portal venopathy, NRH, and ISC [11]. To focus on histologic components of PSVD, clinical signs of portal hypertension were not used for evaluation. The number of cases that would have been classified as PSVD due to the presence of any one (or more) of these histologic lesions, regardless of clinical history, was counted from groups A and B, and compared with Fisher’s exact test. *P* < 0.05 was considered statistically significant.

## Results

### Interobserver study

**Fig. 2:**
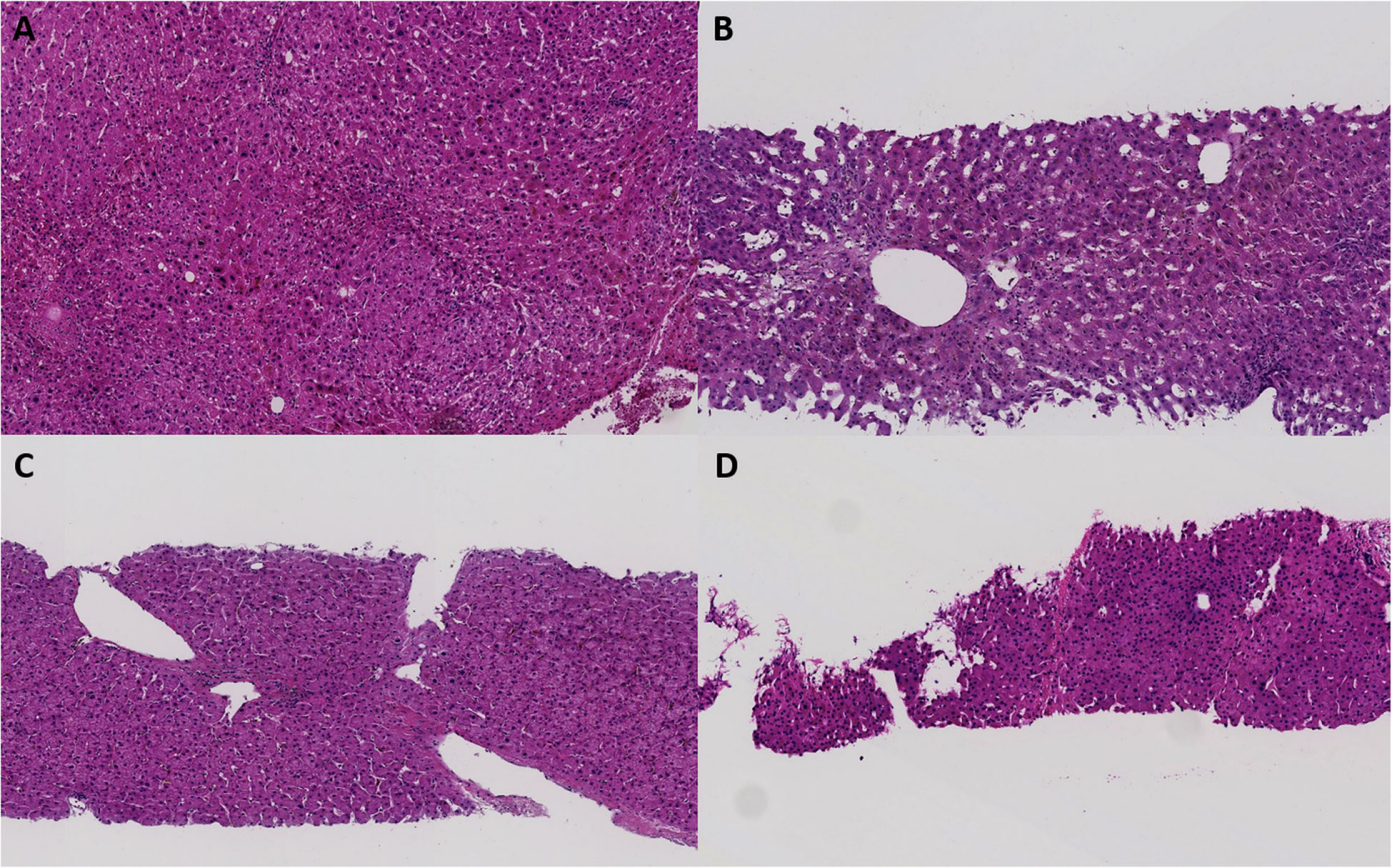
Representative lobular images assessed by pathologists. No consensus was reached on these images on Q1 (no portal hypertension), but consensus was reached on provision of Q2 history (with portal hypertension) as follows: **a**, nodular regeneration; **b**, sinusoidal dilatation; **c**, increased parenchymal draining veins (reprinted by Permission of SAGE Publications, [12], copyright 2015); **d**, incomplete fibrous septa. **a**, **b** and **d** are from idiopathic non-cirrhotic portal hypertension (INCPH) group; **c** is from non-INCPH group [A-D, hematoxylin and eosin, × 100]

**Fig. 3:**
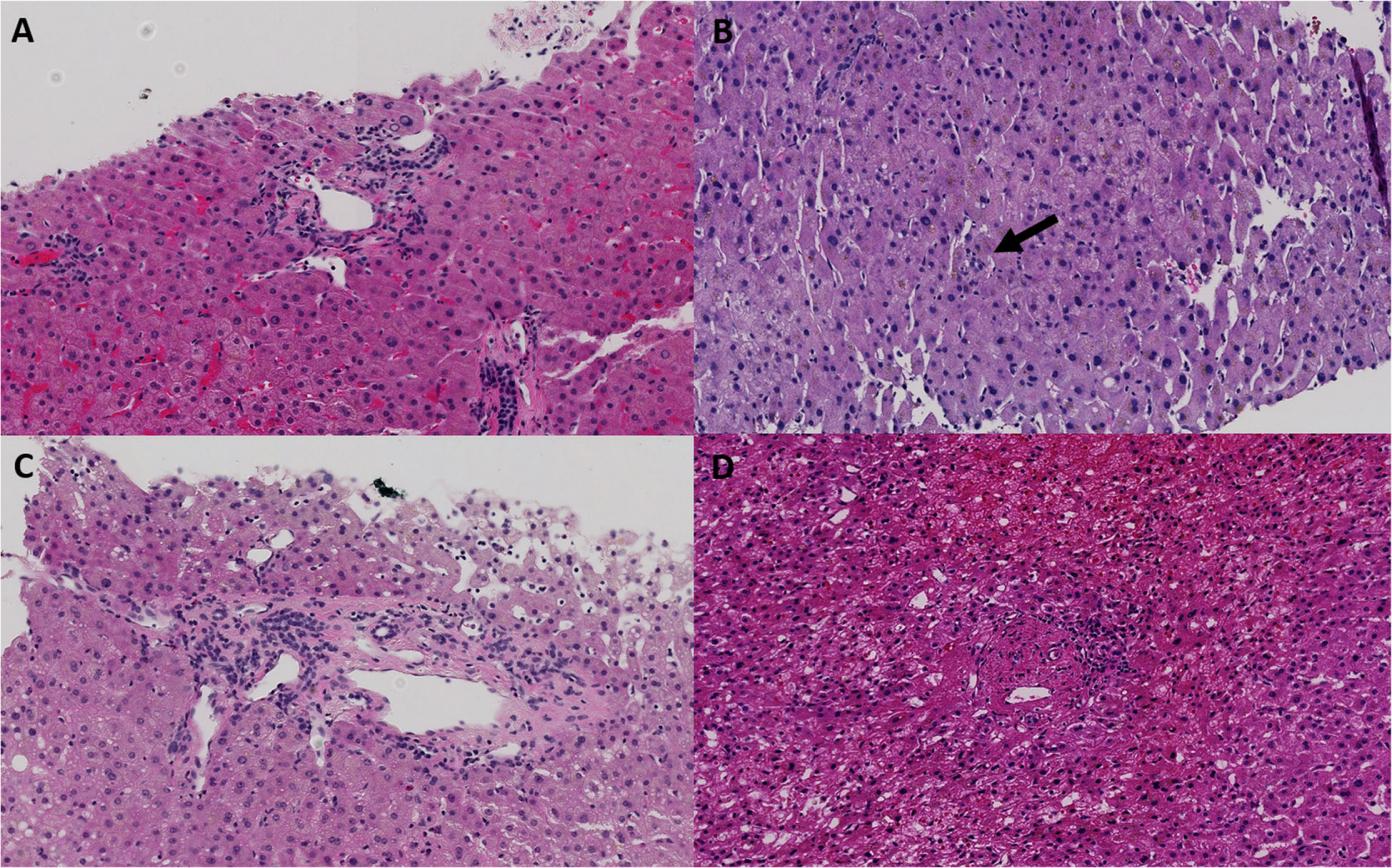
Representative portal tract images assessed by pathologists. No consensus was reached on these images on Q1 (no portal hypertension), but consensus was reached on provision of Q2 history (with portal hypertension) as follows: **a**, paraportal shunting vessels; **b**, portal tract remnant (arrow); **c**, increased number of portal vessels; **d**, obliterative portal venopathy. **d** is from idiopathic non-cirrhotic portal hypertension (INCPH) group; **a-c** are from non-INCPH group [**a**-**d**, hematoxylin and eosin, × 200]

Overall consensus was reached for 60–73% of the images for each category (L-Q1, L-Q2, P-Q1, and P-Q2). Interobserver agreement regarding whether the pathologists considered the image within normal limits (a) or not (non-a) was slight to fair (Table 2). However, when the clinical history of portal hypertension was provided, there were differences in the interpretation of the images with the largest difference in consensus with regard to anisocytosis (− 10), followed by incomplete fibrous septa (+ 6), increased number of portal vessels (+ 5), paraportal shunting vessel(s) (+ 4), and portal tract remnant (+ 3) (Table 2) (Figs. 2 and 3). Four pathologists changed the interpretation based on provided history of portal hypertension. The mean number of images where diagnosis was impacted by clinical history was 40 (range: 10–70 images). However, the impact on the degree of agreement as manifested by the Fleiss’ kappa value was not significantly different between the kappa values for Q1 and Q2.

**Table 2:** Summary of the results

Images distributed		**Lobule (L)** (*n* = 45)		**Portal tract (P)** (*n* = 45)	
Provided clinical history		Q1	Q2	Q1	Q2
Consensus ^a^		73%	67%	60%	69%
Kappa value (95% CI, *p < 0.05*)^b^		0.24 (0.19–0.29)	0.24 (0.19–0.29)	0.18 (0.13–0.23)	0.18 (0.13–0.23)
Change in consensus status by Q2 provision^c^		29%		40%	
WNL to abnormal or loss of consensus, by Q2 provision		9%		16%	
Study group		**INCPH** (L = 25, *P* = 19)		**Non-INCPH** (L = 20, *P* = 26)	
Provided (true) clinical history		Q2		Q1	
Kappa value (95% CI, *p < 0.05*)^b^		L: 0.32 (0.25–0.38)		L: 0.12 (0.05–0.19)	
		P: 0.17 (0.10–0.25)		P: 0.14 (0.08–0.21)	
Reproducibility	Reproducible	57%		37%	
	Non-reproducible	32%		43%	
	Divergent	11%		20%	

Q1: Absence of portal hypertension, Q2: Presence of portal hypertension. ^a^, agreed by at least 6 pathologists; CI, confidence interval; ^b^, unweighted, within normal limits versus abnormal; ^c^, consensus to no consensus or vice versa; INCPH, idiopathic noncirrhotic portal hypertension; WNL, within normal limits by consensus

In group A (INCPH group), the interpretations of within normal limits (*n* = 10, 4 L, 6P), sinusoidal dilatation (*n* = 5), and increased parenchymal draining veins (*n* = 3) were reproducible (i.e., consensus was reached by ≥6 pathologists on both Q1 and Q2), while the interpretations of nodular regeneration and increased number of portal vessels were either non-reproducible (i.e., consensus to no consensus or vice versa) or divergent (i.e., no consensus was reached on both Q1 and Q2). In group B (non-INCPH group), the interpretations of within normal limits (*n* = 8, 2 L, 6P), sinusoidal dilatation (*n* = 4), and paraportal shunting vessel(s) (n = 3) were reproducible, whereas the interpretations of nodular regeneration, incomplete fibrous septa, increased number of portal vessels, and obliterative portal venopathy were either non-reproducible or divergent (Table 2).

### Histologic re-classification of PSVD

When the cases from INCPH (group A) and non-INCPH (group B) were re-assessed for the presence of specific histologic lesions for PSVD (i.e., obliterative portal venopathy, NRH, and ISC), irrespective of clinical history of portal hypertension, 9 of 15 (60%) group A cases and 7 of 19 (37%) group B cases were classified as PSVD (*p* > 0.05) (Additional file 1).

## Discussion

The approach to the diagnosis of INCPH on liver biopsies has been, to a certain extent, conservative. It is well recognized that histologic changes of INCPH can be subtle, patchy, and nonspecific, as they can be seen in a variety of other liver diseases. In fact, Guido et al. emphasized the importance of obtaining clinical history and correlating with imaging before making a diagnosis of INCPH on a liver biopsy [2]. For liver biopsies performed in the setting of abnormal liver function tests of unknown etiology and in the absence of portal hypertension, the presence of histologic findings suggestive of INCPH should be interpreted with caution, but a diagnosis of INCPH could be suggested with the possibility of a preclinical phase of the disease [2]. On the other hand, the newly proposed PSVD classification identifies three specific histologic features (namely, obliterative portal venopathy, NRH, and ISC) which, when present, are sufficient for the diagnosis even in the absence of any clinical or radiological evidence of portal hypertension, as long as the sampling is adequate (liver biopsy ≥20 mm) and cirrhosis is absent [11]. In other words, PSVD is no longer restricted to cases with evidence of portal hypertension but encompasses a broader spectrum of hepatic vascular-type injury pattern [15]. However, as noted before, these histologic features can be seen in non-INCPH/PSVD cases, and their relevance in the diagnosis of INCPH/PSVD remains unknown.

In this regard, our results demonstrate that interobserver agreement on the interpretation of lobular and portal images for INCPH, even when assessed by liver pathologists, is fair at most, regardless of provision of clinical history. Although consensus was achieved for 60–73% of the provided images for each category (L-Q1, L-Q2, P-Q1, and P-Q2), the corresponding kappa values remained relatively unsatisfactory. The level of interobserver agreement did not improve even when true clinical histories were provided for the images although about a half of the participating pathologists changed their assessment based on the provided clinical history. While our group was not in agreement as to whether clinical history should be a factor when assessing whether a feature was present or absent, this appears to reflect the prevailing recognition that clinical context is of utmost importance when evaluating potential INCPH cases.

The most volatile histologic features that were affected by clinical history were anisocytosis, incomplete fibrous septa, and increased number of portal vessels. Likewise, there was no reproducibility for nodular regeneration and increased number of portal vessels in both groups A (INCPH) and B (non-INCPH), and for incomplete fibrous septa and obliterative portal venopathy in group B. These findings seem to suggest that nodular regeneration, incomplete fibrous septa, obliterative portal venopathy, and increased number of portal vessels will likely remain as the most difficult features to recognize consistently in INCPH/PSVD, in which the former three are considered specific for PSVD. In fact, NRH is often regarded as a subtle finding with low interobserver agreement among liver pathologists [16]. Based on our reproducibility study results, a pathologist can consider INCPH as a possibility when sinusoidal dilatation along with the increased number of parenchymal draining veins is recognized, in the right clinical context.

Overall, these results suggest that histologic findings commonly associated with INCPH/PSVD can be seen in ostensibly normal livers and in a broader group of liver disorders. Indeed, after reviewing the cases for the presence of specific histologic features for PSVD (irrespective of signs of portal hypertension), 37% of group B (non-INCPH group) cases were reclassified as PSVD by two authors. This further reinforces the notion that histologic features of INCPH/PSVD are not limited to patients with portal hypertension. Our findings also confirm the previous notion that it may be extremely difficult to make an accurate diagnosis of INCPH without proper clinical context.

Given the marked histologic overlap between INCPH and non-INCPH cases, with at most fair interobserver agreement, it will be challenging to identify a subset of PSVD cases that represent INCPH with associated risk factors, for whom a management implication should be inferred. Also, given that the diagnosis of PSVD primarily relies on recognition of “specific” histologic features, implementation of more well-defined criteria for each histopathologic feature would be needed to improve diagnostic accuracy and reduce interobserver variation. However, the fact that the specific histologic features of PSVD tended to be more common in INCPH (group A) than non-INCPH (group B) (Additional file 1) seems to suggest that there may be some differences in the severity and extent of these histologic changes between the two groups.

The main strength of our study is that 9 liver pathologists with different training backgrounds participated in the study. The fact that the pathologists were blinded to the original diagnosis and clinical history was useful in minimizing any potential observer bias, even though the participants knew that they were involved in an INCPH study. However, there are some limitations to our study as well. For instance, the principal investigator selected non-INCPH cases and the specific areas to be captured digitally in both groups A and B, potentially leading to a selection bias. Although these images still allowed for both overall and focused assessment of lobular and portal tract changes, one may argue that whole digitized or glass slides should have been used for the study. However, we felt that our approach was justified, since histologic changes of INCPH can be very subtle and patchy, and the main objective of our study was to determine interobserver agreement on individual histologic features by having all the participating pathologists examine the exact same areas of lobular and portal tract changes. In support of this, Jharap et al. demonstrated that interobserver agreement on NRH remained poor even when pathologists reviewed the entire slides [16].

## Conclusions

Interpretation of individual histologic features of INCPH is subjected to high degree of interobserver variability and may be influenced by clinical history. Moreover, the histologic features of INCPH are not limited to patients with portal hypertension. With the broadened spectrum of PSVD, histologic assessment of PSVD is likely to remain challenging. Consensus discussion may enhance the diagnostic accuracy of different histologic features that may be encountered within the range of this entity. Most importantly, rigorous clinical validation of those histologic criteria is needed; which will enable us to refine diagnostic criteria and understand clinical significance of those findings.

## Supplementary information

Additional file 1. Specific histologic lesions of Porto-sinusoidal vascular disease (PSVD) in idiopathic noncirrhotic portal hypertension (INCPH) and non-INCPH. All INCPH patients had portal hypertension. OPV, obliterative portal venopathy; NRH, nodular regenerative hyperplasia; ISC, incomplete septal cirrhosis. (DOCX 14.3 kb)

## Data Availability

All data generated or analysed during this study are included in this published article and its supplementary file.

## Abbreviations

INCPH: Idiopathic non-cirrhotic portal hypertension
PSVD: Porto-sinusoidal vascular disease
NRH: Nodular regenerative hyperplasia
ISC: Incomplete septal cirrhosis

## References

[1] Lee H, Rehman A, Fiel MI (2016). Idiopathic noncirrhotic portal hypertension: an appraisal. J Pathol Transl Med.

[2] Guido M, Sarcognato S, Sacchi D, Colloredo G (2018). Pathology of idiopathic non-cirrhotic portal hypertension. Virchows Arch.

[3] Schouten JN, Garcia-Pagan JC, Valla DC, Janssen HLA (2011). Idiopathic noncirrhotic portal hypertension. Hepatology..

[4] Wanless IR, Godwin TA, Allen F, Feder A (1980). Nodular regenerative hyperplasia of the liver in hematologic disorders: a possible response to obliterative portal venopathy. A morphometric study of nine cases with an hypothesis on the pathogenesis. Medicine (Baltimore).

[5] Rajekar H, Vasishta RK, Chawla YK, Dhiman RK (2011). Noncirrhotic portal hypertension. J Clin Exp Hepatol.

[6] Schouten JN, Verheij J, Seijo S (2015). Idiopathic non-cirrhotic portal hypertension: a review. Orphanet J Rare Dis.

[7] Hillaire S, Bonte E, Denninger MH (2002). Idiopathic non-cirrhotic intrahepatic portal hypertension in the west: a re-evaluation in 28 patients. Gut..

[8] Zuo C, Chumbalkar V, Ells PF, Bonville DJ, Lee H (2017). Prevalence of histological features of idiopathic noncirrhotic portal hypertension in general population: a retrospective study of incidental liver biopsies. Hepatol Int.

[9] El Jabbour T, McHugh KE, Patil DT, Zuo C, Koo BH, Kim S, Lee H (2020). Histologic lesions of Porto-Sinusoidal Vascular Disease following phlebotomy in hemochromatosis. Gastroenterol Res.

[10] Nakanuma Y, Hoso M, Sasaki M (1996). Histopathology of the liver in non-cirrhotic portal hypertension of unknown aetiology. Histopathology..

[11] De Gottardi A, Rautou PE, Schouten J (2019). Porto-sinusoidal vascular disease: proposal and description of a novel entity. Lancet Gastroenterol Hepatol.

[12] Lee H, Ainechi S, Singh M (2015). Histologic spectrum of idiopathic noncirrhotic portal hypertension in liver biopsies from dialysis patients. Int J Surg Pathol.

[13] Verheij J, Schouten JN, Komuta M, Nevens F, Hansen BE, Janssen HL, Roskams T (2013). Histologic features in western patients with idiopathic non-cirrhotic portal hypertension. Histopathology..

[14] Crawford AR, Lin X, Crawford JM (1998). The normal adult human liver biopsy: a quantitative reference standard. Hepatology..

[15] Gioia S, Nardelli S, Ridola L, d'Amati G, Riggio O (2019). Is Porto sinusoidal vascular disease to be actively searched in patients with portal vein thrombosis?. World J Hepatol.

[16] Jharap B, van Asseldonk DP, de Boer NK (2015). Diagnosing nodular regenerative hyperplasia of the liver is thwarted by low interobserver agreement. PLoS One.

