# Supplementary material for: Interobserver study on histologic features of idiopathic non-cirrhotic portal hypertension
Source: Diagn Pathol. 2020 Oct 23;15:129. doi: 10.1186/s13000-020-01049-0 (PMC7583235; doi:10.1186/s13000-020-01049-0)
Supplement: Supplementary file 1 — Specific histologic lesions of Porto-sinusoidal vascular disease (PSVD) in idiopathic noncirrhotic portal hypertension (INCPH) and non-INCPH. All INCPH patients had portal hypertension. OPV, obliterative portal venopathy; NRH, nodular regenerative hyperplasia; ISC, incomplete septal cirrhosis. (DOCX 14.3 kb) [file 13000_2020_1049_MOESM1_ESM.docx]

| Case (INCPH) | Specific histologic lesions of PSVD | Case (non-INCPH) | Specific histologic lesions of PSVD |
| --- | --- | --- | --- |
| 1 | OPV | 1 | ISC |
| 2 | - | 2 | OPV |
| 3 | - | 3 | - |
| 4 | OPV, ISC | 4 | - |
| 5 | OPV, NRH | 5 | - |
| 6 | - | 6 | - |
| 7 | OPV, NRH | 7 | - |
| 8 | OPV | 8 | OPV |
| 9 | OPV, ISC | 9 | OPV |
| 10 | NRH | 10 | - |
| 11 | - | 11 | - |
| 12 | - | 12 | - |
| 13 | OPV, NRH | 13 | - |
| 14 | OPV, NRH | 14 | - |
| 15 | - | 15 | OPV |
|  |  | 16 | OPV |
|  |  | 17 | - |
|  |  | 18 | OPV |
|  |  | 19 | - |

Additional file 1. Specific histologic lesions of Porto-sinusoidal vascular disease (PSVD) in idiopathic noncirrhotic portal hypertension (INCPH) and non-INCPH. All INCPH patients had portal hypertension. OPV, obliterative portal venopathy; NRH, nodular regenerative hyperplasia; ISC, incomplete septal cirrhosis.
